# Dental Anxiety in Periodontal Patients attending a University Clinic in northeastern Mexico

**DOI:** 10.21142/2523-2754-1404-2026-313

**Published:** 2026-10-01

**Authors:** José L. Jiménez-Ortiz, Guillermo Cano-Verdugo, Josué D. Jiménez-Ortiz, Rosa M. Islas-Valle, Benjamín Tello-García, Luis R. Sosa-Martínez, Myriam A de la Garza-Ramos, Adrián Sesma-Pereyra

**Affiliations:** 1 Montemorelos University School of Dentistry. Montemorelos, Nuevo León, México. jimenezortiz@um.edu.mx btello@um.edu.mx rsosa@um.edu.mx Montemorelos University School of Dentistry Montemorelos Nuevo León México jimenezortiz@um.edu.mx btello@um.edu.mx rsosa@um.edu.mx; 2 Autonomous University of Nuevo León, Faculty of Public Health and Nutrition. Monterrey, Nuevo León, México. guillermo.canovrd@uanl.edu.mx Autonomous University of Nuevo León Faculty of Public Health and Nutrition Monterrey Nuevo León México guillermo.canovrd@uanl.edu.mx; 3 Mexican Social Security Institute, Family Medical Unit N.° 1. Obregón, Sonora, México. dr.jimenezortiz@gmail.com Mexican Social Security Institute Family Medical Unit N.° 1 Obregón Sonora México dr.jimenezortiz@gmail.com; 4 University of Montemorelos, Faculty of Health Sciences. Montemorelos, Nuevo León, México. roziizlaz@gmail.com University of Montemorelos Faculty of Health Sciences Montemorelos Nuevo León México roziizlaz@gmail.com; 5 Autonomous University of Nuevo León, Faculty of Dentistry. Monterrey, Nuevo León, México. myriam.garzarm@uanl.edu.mx Autonomous University of Nuevo León Faculty of Dentistry Monterrey Nuevo León México; 6 University of Sciences and Arts of Chiapas, Faculty of Dental Sciences and Public Health. Tuxtla Gutiérrez, Chiapas, México. adrian.sesma@unicach.mx University of Sciences and Arts of Chiapas Faculty of Dental Sciences and Public Health Tuxtla Gutiérrez Chiapas México adrian.sesma@unicach.mx

**Keywords:** dental anxiety, periodontics, oral health, dental clinic, Mexico, ansiedad dental, periodoncia, salud bucal, clínica dental, México

## Abstract

**Objective:**

To determine the level of dental anxiety in adult patients attending periodontal evaluation at a university dental clinic in northeastern Mexico.

**Materials and Methods:**

A cross-sectional study was conducted using a non-probabilistic convenience sample of adult patients (≥18 years). Dental anxiety was assessed prior to periodontal evaluation using the Spanish version of the Short Dental Anxiety Inventory (S-DAI). Sociodemographic variables were recorded. Descriptive statistics were calculated, and associations were evaluated using chi-square tests (p < 0.05).

**Results:**

A total of 189 participants were included (mean age 38.53 ± 16.38 years). Overall, 67.6% of participants presented some level of dental anxiety. Mild anxiety was the most frequent category (37.6%), followed by no anxiety (32.3%), moderate anxiety (12.7%), and severe anxiety (17.5%). Statistically significant associations were found between dental anxiety and sex (p = .002) and occupation (p = .035), while no significant associations were observed for age, place of residence, marital status, or educational level.

**Conclusions:**

Dental anxiety is highly prevalent among patients undergoing periodontal evaluation. The use of brief, validated instruments such as the S-DAI during the initial consultation may facilitate early identification of anxious patients and support the implementation of patient-centered strategies to improve treatment adherence.

## RESUMEN

**Objetivo:**

Determinar el nivel de ansiedad dental en pacientes adultos que acuden a evaluación periodontal en una clínica odontológica universitaria del noreste de México.

**Materiales y métodos:**

Se realizó un estudio transversal con una muestra no probabilística por conveniencia de pacientes adultos (≥18 años). La ansiedad dental se evaluó previo a la consulta periodontal mediante la versión en español del *Short Dental Anxiety Inventory* (S-DAI). Se registraron variables sociodemográficas. Se calcularon estadísticas descriptivas y se evaluaron asociaciones mediante la prueba de chi-cuadrado (p < 0.05).

**Resultados:**

Se incluyeron 189 participantes (edad media 38.53 ± 16.38 años). En total, el 67.6% presentó algún nivel de ansiedad dental. La ansiedad leve fue la categoría más frecuente (37.6%), seguida de ausencia de ansiedad (32.3%), ansiedad moderada (12.7%) y ansiedad severa (17.5%). Se encontraron asociaciones estadísticamente significativas entre la ansiedad dental y el sexo (p = .002) y la ocupación (p = .035), mientras que no se observaron asociaciones significativas con la edad, lugar de residencia, estado civil ni nivel educativo.

**Conclusiones:**

La ansiedad dental presenta una alta prevalencia en pacientes que acuden a evaluación periodontal. El uso de instrumentos breves y validados como el S-DAI durante la consulta inicial puede facilitar la identificación temprana de pacientes con ansiedad y apoyar la implementación de estrategias centradas en el paciente para mejorar la adherencia al tratamiento.

## INTRODUCTION

Dental anxiety is a prevalent psychological condition characterized by excessive fear or stress associated with dental care, often leading to avoidance behaviors and negative oral health outcomes ^1^^-^^3^. It is estimated that between 10% and 20% of the global population experiences moderate to severe dental anxiety, with higher prevalence among individuals requiring invasive dental procedures ^4^, ^5^. This condition has important clinical implications, as it contributes to delayed dental attendance, poor oral hygiene practices, and increased disease burden, including caries and periodontal disease ^6^^,^^7^. Additionally, dental anxiety has been associated with psychological and physiological responses such as increased stress, altered pain perception, and reduced treatment adherence ^8^^-^^10^.

Periodontal disease is a chronic inflammatory condition with high global prevalence, affecting a substantial proportion of adults worldwide ^11^^,^^12^. It is characterized by complex interactions between microbial biofilm and host immune-inflammatory responses, leading to progressive destruction of the supporting structures of the teeth ^13^. In Mexico, periodontal diseases represent a major public health concern, with a high prevalence of gingivitis and periodontitis reported in national epidemiological surveillance systems ^14^. The chronic nature of periodontal disease, along with the need for invasive diagnostic and therapeutic procedures, may increase anticipatory anxiety in affected patients.

Despite the recognized relationship between anxiety and oral health, most studies have focused on general dental populations. Patients requiring periodontal care may represent a distinct subgroup due to the complexity and perceived invasiveness of their treatment needs. Furthermore, university dental clinics provide care in a unique context, often involving treatment by students under supervision and serving populations with diverse socioeconomic backgrounds, which may influence patients’ perceptions and anxiety levels. In Mexico, particularly in the northern region, there is limited evidence addressing dental anxiety specifically in periodontal patients within university clinical settings. Understanding anxiety levels in this population is essential to improve patient-centered care and treatment adherence. Therefore, the aim of this study was to determine the level of dental anxiety in adult patients attending periodontal evaluation at a university dental clinic in northeastern Mexico.

## MATERIAL AND METHODS

### Study design and setting

A cross-sectional study was conducted at the Dental Clinic of Montemorelos University, located in Nuevo León, Mexico, between August and December 2019. The clinic provides care primarily to patients requiring comprehensive dental and periodontal treatment.

### Population and sampling

The study population consisted of adult patients attending the university dental clinic for periodontal evaluation. A non-probabilistic convenience sampling approach was used, including all eligible patients who attended the clinic during the study period. Participants were included if they were aged 18 years or older, of either sex, and agreed to participate voluntarily by providing informed consent. Patients who attended the clinic for emergency care, those who did not complete at least 95% of the questionnaire items, and those who declined participation were excluded. A total of 197 patients were initially assessed for eligibility. Of these, six were excluded for not meeting the inclusion criteria and two declined participation, resulting in a final sample of 189 participants included in the analysis. The participant selection process is summarized in a flow diagram (Figure 1). Given the exploratory nature of this study and the absence of prior local data on dental anxiety in periodontal patients in this specific setting, no formal sample size calculation was performed. This approach is consistent with observational studies designed to generate preliminary evidence in underexplored populations.

**Figure 1:**
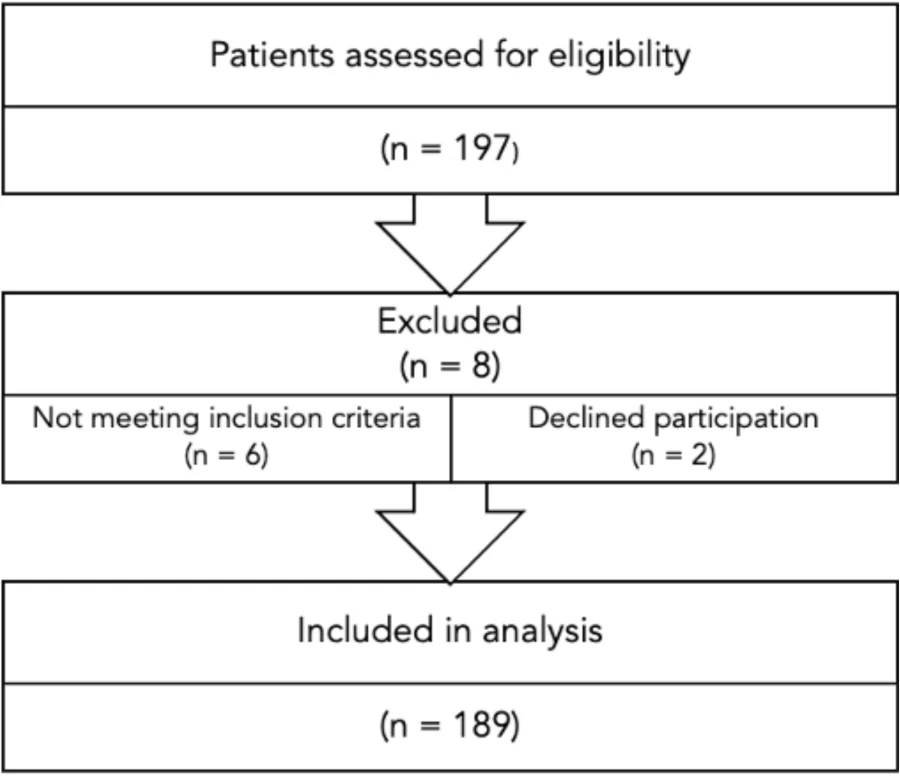
Flow diagram of participant selection process.

### Timing of assessment

Dental anxiety was assessed prior to periodontal evaluation, before any diagnostic or therapeutic procedures were performed. This ensured that anxiety levels reflected anticipatory responses rather than reactions to clinical interventions.

### Instrument and variables

Dental anxiety was measured using the Spanish version of the Short Version of the Dental Anxiety Inventory (S-DAI), previously validated in Mexican adult patients ^15^. The instrument consists of nine items that assess anxiety-related responses in dental care situations. Each item is scored on a Likert scale ranging from “never” to “always.” The total score is obtained by summing the responses, and anxiety levels were categorized into four levels based on predefined cut-off points. In this study, the internal consistency of the instrument was evaluated using Cronbach’s alpha, yielding a value of 0.92, indicating excellent reliability. Sociodemographic variables included age (in years), sex (male/female), place of residence (urban/rural), marital status, educational level, and occupation.

### Data collection procedure

Data collection was performed through face-to-face interviews conducted by five previously trained dental professionals, ensuring standardized administration of the instrument. The questionnaire was applied in a single session in the clinic’s consultation area, with an average completion time of approximately 15 minutes. All completed questionnaires were stored securely for subsequent data processing.

### Statistical analysis

Data were analyzed using IBM SPSS Statistics software (version 25). Descriptive statistics were calculated, including measures of central tendency and dispersion for continuous variables and frequencies and percentages for categorical variables. Associations between dental anxiety levels and sociodemographic variables were evaluated using the chi-square test. Prior to analysis, assumptions for the chi-square test were assessed, and categories were collapsed where necessary to ensure that expected cell counts met the assumptions required for chi-square analysis. A significance level of p < 0.05 was established for all inferential analyses.

### Ethical considerations

All procedures were conducted in accordance with the ethical standards of the Declaration of Helsinki and the Mexican regulations for research involving human subjects (NOM-012-SSA3-2012). The study protocol was approved by the Ethics Committee of Montemorelos University (Approval ID: 2019-060). Written informed consent was obtained from all participants prior to their inclusion in the study.

### Reporting standards

This study adhered to the Strengthening the Reporting of Observational Studies in Epidemiology (STROBE) guidelines ^16^.

## RESULTS

### Sociodemographic characteristics

A total of 189 participants were included in the analysis, with a mean age of 38.53 ± 16.38 years. The sample was almost evenly distributed by sex (51% male, 49% female), with the majority residing in urban areas (75%). Most participants were single (46%) or married (41%), and the most frequent educational level was high school (35.4%). Regarding occupation, employees (32.8%) and students (22.2%) were the most represented groups (Table 1).

**Table 1:** Sociodemographic characteristics of the study population (n = 189)

Variable	Category	n (%)
Age (years)	Mean ± SD	38.53 ± 16.38
Sex	Male	97 (51.3)
	Female	92 (48.7)
Place of residence	Urban	142 (75.1)
	Rural	47 (24.9)
Marital status	Single	88 (46.6)
	Married	78 (41.3)
	Widowed	9 (4.8)
	Common law	9 (4.8)
	Divorced	5 (2.6)
Education level	High school	67 (35.4)
	Bachelor’s degree	50 (26.5)
	Postgraduate	23 (12.2)
	Other*	49 (25.9)
Occupation	Employee	62 (32.8)
	Student	42 (22.2)
	Unemployed	48 (25.4)
	Other*	37 (19.6)

*Other categories include participants with educational or occupational characteristics not classified in the predefined groups.

### Distribution of dental anxiety levels

Overall, 67.6% of participants presented some level of dental anxiety. Mild anxiety was the most frequent category (37.6%), followed by no anxiety (32.3%), moderate anxiety (12.7%), and severe anxiety (17.5%) (Figure 2).

**Figure 2:**
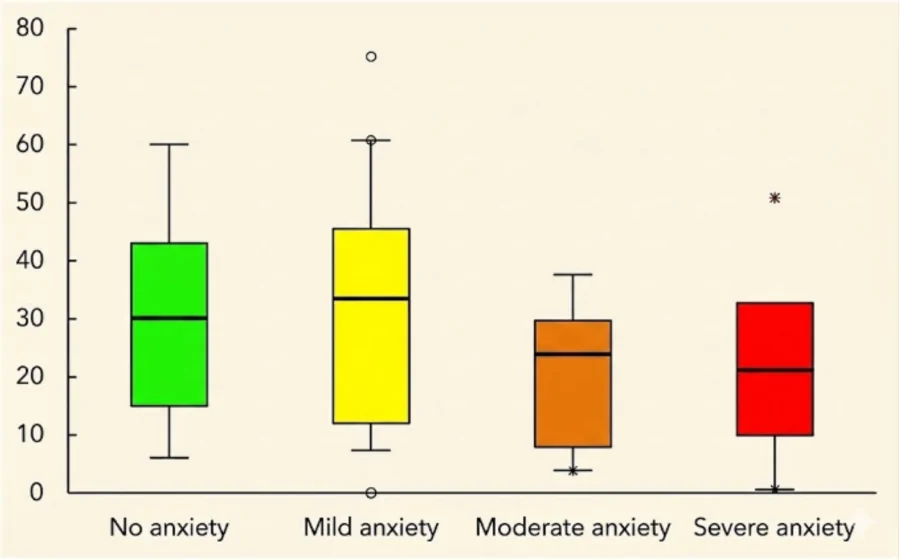
Distribution of dental anxiety levels among participants.

### Association between dental anxiety and sociodemographic variables

To meet the assumptions required for chi-square analysis, anxiety categories were grouped into three levels (no anxiety, mild anxiety, and moderate-severe anxiety). Among the sociodemographic variables analyzed, only sex and occupation showed statistically significant associations with dental anxiety levels (p < 0.05) (Table 2). Women exhibited a higher proportion of moderate-severe anxiety compared to men, whereas men were more frequently classified as having no anxiety. Regarding occupation, retired individuals and homemakers showed higher proportions of moderate-severe anxiety, while students and employees were more commonly classified within the no anxiety or mild anxiety categories. No statistically significant associations were observed for age, place of residence, marital status, or educational level.

**Table 2:** Association between dental anxiety levels and sociodemographic variables (n = 189)

Variable	Category	No anxiety n (%)	Mild anxiety n (%)	Moderate-Severe anxiety n (%)	p-value
Sex	Male	38 (62.3)	39 (54.9)	20 (35.1)	0.002
	Female	23 (37.7)	32 (45.1)	37 (64.9)	
Place of residence	Urban	49 (80.3)	48 (67.6)	45 (78.9)	0.281
	Rural	12 (19.7)	23 (32.4)	12 (21.1)	
Marital status	Single	32 (52.5)	33 (46.5)	23 (40.4)	0.663
	Married/Other*	29 (47.5)	38 (53.5)	34 (59.6)	
Education level	≤ High school	43 (70.5)	46 (64.8)	29 (50.9)	0.620
	> High school	18 (29.5)	25 (35.2)	28 (49.1)	
Occupation	Employee/Student	38 (62.3)	38 (53.5)	18 (31.6)	0.035
	Other**	23 (37.7)	33 (46.5)	39 (68.4)	

*Includes married, widowed, divorced, and common law.

**Includes unemployed, homemakers, retired, and other occupations. Moderate and severe anxiety categories were grouped to meet the assumptions required for chi-square analysis. Chi-square test was used to assess associations. Statistical significance was set at p < 0.05. Percentages are presented by row.

### Item-level analysis of dental anxiety

At the item level, the highest anxiety responses were associated with invasive or sensory-related stimuli, particularly the sound of the dental drill and the anticipation of tooth extraction. The item referring to the sound of the drilling machine presented the highest mean score. In contrast, the lowest levels of anxiety were observed in items related to physiological reactions in the waiting room, such as sweating or trembling, which showed the lowest mean values. Detailed response distributions for each item are presented in Table 3.

**Table 3:** Distribution of responses for each item of the Dental Anxiety Inventory (S-DAI) (n = 189)

Item	Always n (%)	Almost always n (%)	Sometimes n (%)	Seldom n (%)	Never n (%)	Mean	SD
1. Tooth extraction anxiety	28 (14.8)	12 (6.3)	33 (17.5)	13 (6.9)	103 (54.5)	2.20	1.50
2. Anxiety about drilling	26 (13.8)	8 (4.2)	25 (13.2)	16 (8.5)	114 (60.3)	2.03	1.46
3. Anxiety before sitting in chair	27 (14.3)	11 (5.8)	22 (11.6)	16 (8.5)	113 (59.8)	2.06	1.49
4. Lack of control in chair	13 (6.9)	11 (5.8)	14 (7.4)	20 (10.6)	131 (69.3)	1.70	1.24
5. Sound of drill	36 (19.0)	15 (7.9)	33 (17.5)	14 (7.4)	91 (48.1)	2.42	1.58
6. Physical reaction in waiting room	11 (5.8)	8 (4.2)	14 (7.4)	16 (8.5)	140 (74.1)	1.59	1.16
7. Anxiety to anesthetic needle	18 (9.5)	9 (4.8)	17 (9.0)	20 (10.6)	125 (66.1)	1.81	1.33
8. Nervous when invited to chair	22 (11.6)	12 (6.3)	18 (9.5)	20 (10.6)	117 (61.9)	1.95	1.42
9. Fear of lack of explanation	16 (8.5)	11 (5.8)	17 (9.0)	17 (9.0)	128 (67.7)	1.78	1.31

Note: Responses were recorded using a five-point Likert scale ranging from “never” to “always”. Values are presented as frequency (percentage), mean, and standard deviation.

## DISCUSSION

The present study aimed to determine the level of dental anxiety among adult patients attending a periodontal evaluation at a university dental clinic in northeastern Mexico. The findings revealed that approximately two-thirds of participants (67.6%) experienced some level of dental anxiety, with nearly one-third presenting moderate to severe anxiety levels. Additionally, sex and occupation were the only sociodemographic variables significantly associated with dental anxiety. These results highlight that dental anxiety is highly prevalent in patients seeking periodontal care, reinforcing its relevance as a clinical and public health concern in this population.

The proportion of patients with moderate to severe anxiety observed in this study is consistent with previous reports conducted in dental clinical settings, where similar prevalence ranges have been described ^4^^,^^17^^,^^18^. Furthermore, studies conducted in Mexican populations attending university dental clinics have also reported moderate to high levels of dental fear and anxiety ^19^^,^^20^, highlighting the persistence of this phenomenon in similar clinical and educational contexts. This supports the notion that patients requiring periodontal care may experience higher levels of anxiety, likely due to the perceived invasiveness and chronic nature of periodontal treatment. However, differences have been observed regarding associated factors; while some studies in Mexican populations have not identified significant associations between sex and dental anxiety ^19^, our findings indicate that women presented higher levels of severe anxiety. This aligns with reports from other international contexts ^21^, where gender differences have been attributed to psychosocial and cultural factors influencing emotional expression and perception of dental care. Regarding occupation, the observed association suggests that socioeconomic and lifestyle factors may play a role in shaping anxiety responses. Individuals such as homemakers and retired participants exhibited higher anxiety levels, which may be related to increased exposure to stressors, limited access to oral health information, or previous negative dental experiences.

The high prevalence of dental anxiety observed in this study can be understood within a biopsychosocial framework. Periodontal patients often require complex and sometimes invasive procedures, which may increase anticipatory anxiety. Additionally, prior negative experiences, fear of pain, and uncertainty about treatment outcomes are well-documented contributors to dental anxiety ^2^^,^^3^^,^^22^. The item-level findings further support this interpretation, as the highest anxiety levels were associated with stimuli such as the sound of the dental drill and tooth extraction ^8^^,^^10^, both commonly linked to pain perception and loss of control. These sensory and cognitive triggers are known to activate fear responses and avoidance behaviors, as well as broader psychosocial impacts ^9^. Moreover, dental anxiety may contribute to a cyclical relationship with periodontal disease. Individuals with higher anxiety levels may delay seeking care, leading to disease progression and the need for more invasive treatments, which in turn reinforce anxiety. This cycle has important implications for both clinical management and long-term oral health outcomes.

The findings of this study underscore the importance of incorporating anxiety assessment into routine periodontal evaluation. The use of brief, validated instruments such as the S-DAI may facilitate early identification of anxious patients and support the implementation of tailored management strategies. From a clinical perspective, interventions such as effective communication, patient education, behavioral techniques, and anxiety-reduction protocols could improve patient experience and treatment adherence ^23^^,^^24^. From a public health standpoint, addressing dental anxiety may contribute to reducing barriers to care and improving overall oral health outcomes in populations with high periodontal disease burden.

One of the main strengths of this study is its focus on specific clinical population-patients requiring periodontal care-which has been less explored in the context of dental anxiety. Additionally, the use of a validated instrument with high internal consistency strengthens the reliability of the findings. However, several limitations should be considered. The use of a non-probabilistic sample from a single university clinic limits the generalizability of the results. The cross-sectional design precludes establishing causal relationships between variables. Furthermore, although chi-square tests were used to explore associations, some categories presented low expected frequencies, which may affect the robustness of certain comparisons. Additionally, relevant psychosocial variables such as previous dental experiences, perceived pain, and psychological stress were not assessed and could have influenced the results.

Future studies should explore dental anxiety in periodontal patients across different clinical settings and populations to enhance external validity. Longitudinal designs would be valuable to assess changes in anxiety throughout the course of periodontal treatment. Moreover, incorporating psychosocial and behavioral variables could provide a more comprehensive understanding of the determinants of dental anxiety. Qualitative approaches may also offer deeper insights into patient experiences and perceptions related to periodontal care.

## CONCLUSIONS 

The findings of this study indicate that dental anxiety is highly prevalent among adult patients attending periodontal evaluation in a university dental clinic in northeastern Mexico, with approximately two-thirds of participants experiencing some level of anxiety and a considerable proportion presenting moderate to severe levels. Among the variables analyzed, only sex and occupation were significantly associated with dental anxiety, suggesting that both demographic and socioeconomic factors may influence patients’ emotional responses to periodontal care. The assessment of dental anxiety during the initial periodontal consultation, using a brief and reliable instrument such as the S-DAI, represents a practical and accessible strategy to identify patients at risk of elevated anxiety. Incorporating this evaluation into routine clinical practice may support the implementation of patient-centered approaches, improve treatment adherence, and contribute to more comprehensive periodontal care.
